# Chemically Defined Medium Enables GDNF-Driven Early Neuronal-like Phenotype of Human Dental Pulp Stem Cells

**DOI:** 10.3390/cells15100953

**Published:** 2026-05-21

**Authors:** Maria-del-Carmen Silva-Lucero, Gustavo Lopez-Toledo, Víctor-Adrián Cortés-Morales, Juan-José Montesinos, Raúl Sampieri-Cabrera, David-E. García, Juan-Ramon Padilla-Mendoza, Obed-Ricardo Lora-Marin, Jesus-Adrian Buendia-Meraz, Fausto-Alejandro Jiménez-Orozco, Israel López-Reyes, Paul Mondragon-Teran, Maria-del-Carmen Cardenas-Aguayo

**Affiliations:** 1 Laboratory of Cellular Reprogramming and Chronic-Degenerative Diseases, Departamento de Fisiología, Facultad de Medicina, Universidad Nacional Autónoma de México (UNAM), Av. Universidad 3000, Coyoacan, Ciudad de México 04510, Mexico; carmenaguila10@hotmail.com (M.-d.-C.S.-L.); glopez@facmed.unam.mx (G.L.-T.); ibtramon.padilla@outlook.com (J.-R.P.-M.); rk69@ciencias.unam.mx (O.-R.L.-M.); adrianuam1030@hotmail.com (J.-A.B.-M.); 2 Laboratoy of Biotechnology, Departamento de Fisiología, Facultad de Medicina, Universidad Nacional Autónoma de México (UNAM), Av. Universidad 3000, Coyoacán, Ciudad de México 04510, Mexico; 3 Laboratory of Neurobiology of Aging, Departamento de Fisiología, Facultad de Medicina, Universidad Nacional Autónoma de México (UNAM), Av. Universidad 3000, Ciudad de México 04510, Mexico; 4 Mesenchymal Stem Cells Laboratory, Oncology Research Unit, Oncology Hospital, Centro Médico Nacional Siglo XXI (IMSS), Ciudad de México 06720, Mexico; v.adrian.cortes@gmail.com (V.-A.C.-M.); montesinosster@gmail.com (J.-J.M.); 5 Unidad de Investigación Médica en Inmunoquímica (UMAE), Hospital de Especialidades, Centro Médico Nacional Siglo XXI (IMSS), Ciudad de México 06720, Mexico; 6 Laboratory of Prospective, Departamento de Fisiología, Facultad de Medicina, Universidad Nacional Autónoma de México (UNAM), Av. Universidad 3000, Coyoacán, Ciudad de México 04510, Mexico; sampieri@comunidad.unam.mx; 7 Laboratory of Membrane and Stem Cell Biophysics, Departamento de Fisiología, Facultad de Medicina, Universidad Nacional Autónoma de México (UNAM), Av. Universidad 3000, Coyoacán, Ciudad de México 04510, Mexico; erasmo@unam.mx; 8 Posgrado en Ciencias (Neurobiología), Universidad Nacional Autónoma de México (UNAM), Boulevard Juriquilla 3001, Juriquilla, Santiago de Querétaro C.P. 76230, Mexico; 9 Laboratory of Cellular Pharmacology, Department of Pharmacology, Faculty of Medicine, Universidad Nacional Autónoma de México (UNAM), Av. Universidad 3000, Coyoacán, Ciudad de México 04510, Mexico; alejo@facmed.unam.mx; 10 Colegio de Ciencias y Humanidades, Universidad Autónoma de la Ciudad de México (UACM), Plantel Cuautepec, Ciudad de México 07160, Mexico; israel.lopez.reyes@uacm.edu.mx; 11 Centro de Investigación en Ciencia Aplicada y Tecnología Avanzada, CICATA-Unidad Morelos, Instituto Politécnico Nacional, Xochitepec 62790, Morelos, Mexico; p.mondragonteran@gmail.com

**Keywords:** chemically defined medium, dental pulp stem cells, hDPSC, neuronal differentiation protowards GDNF, mesenchymal stem cells, electrophysiology, Mexican donor

## Abstract

Background: Human dental pulp stem cells (hDPSCs) are a promising source of multipotent mesenchymal stem cells (MSCs) for regenerative neurology because of their inherent neurogenic potential. However, robust and reproducible protocols for driving their terminal neuronal maturation in a fully defined, xeno-free environment are lacking. Methods: hDPSCs were isolated from a donor tooth and characterized for mesenchymal (CD105, CD90, CD73, CD13) and stemness-associated markers (SOX2, Oct3/4 and Nanog). Cells were differentiated in a novel, fully chemically defined medium 1% ITS medium (ITS: Insulin, Transferrin, Selenium) supplemented with glial cell line-derived neurotrophic factor (GDNF) or brain-derived neurotrophic factor (BDNF). Neuronal commitment and partial maturation were assessed via immunofluorescence, Western blot, and RT-PCR for markers such as NeuN (Neuronal nuclei) and NF-M (Neurofilament medium chain), and functionally by whole-cell patch-clamp electrophysiology. Results: Although undifferentiated hDPSCs expressed neural progenitor markers (βIII-tubulin and Nestin), only GDNF treatment in a chemically defined medium significantly upregulated mature neuronal markers (NeuN and NF-M) and downregulated mesenchymal markers. Importantly, GDNF-treated cells exhibited key functional changes, including hyperpolarized resting membrane potentials, increased membrane capacitance, and elevated input resistance, which are electrophysiological hallmarks of neural precursor or early neuronal maturation, compared to control cells cultured in medium containing fetal bovine serum (FBS). Although action potentials were not elicited, this represents a significant advancement toward achieving a functional neuronal state. Conclusion: This study demonstrates that a fully chemically defined medium enables GDNF to drive hDPSCs beyond the neural progenitor state towards a partially mature neuronal phenotype. This defined medium protocol eliminates serum variability, enhances reproducibility, and provides a critical step towards standardizing hDPSC-derived neuronal cells for disease modeling and cell-based therapy.

## 1. Introduction

The regenerative capacity of the mammalian central nervous system (CNS) is limited, rendering it vulnerable to irreversible damage from traumatic injury or neurodegenerative pathologies, such as Alzheimer’s disease, culminating in progressive neuronal loss and permanent neurological deficits [1,2]. Current pharmacological interventions are primarily palliative, aimed at symptom mitigation rather than facilitating tissue restoration or arresting (delaying) pathogenesis [3]. Consequently, investigative efforts have pivoted towards identifying autologous stem cell sources capable of neural differentiation and neurotrophic support [4].

In this context, human dental pulp stem cells (hDPSCs) have emerged as compelling candidates. They are mesenchymal stem cells (MSCs) that are routinely accessible via minimally invasive extraction of third molars [5], and their isolation may present fewer ethical controversies than their pluripotent counterparts [6]. A key advantage is their robust secretome, which includes anti-inflammatory cytokines [7] and neurotrophic factors like BDNF (Brain-derived neurotrophic factor) and NT-3 (Neurotrophin-3) [8], suggesting a dual therapeutic modality: potential tissue replacement and modulation of the deleterious inflammatory microenvironment at CNS lesions [9]. hDPSCs are of neural crest origin, which underpins their neurogenic potential [6,10].

hDPSCs are a population of mesenchymal stem cells (MSCs) [5]. The International Society for Cell & Gene Therapy (ISCT) has established minimal criteria for MSCs, including adherence to plastic, specific surface marker expression (CD73, CD90, and CD105; lacking CD45, CD34, CD14, and HLA-DR), and trilineage differentiation potential [11,12]. MSCs are present in many adult tissues, including the bone marrow [13], adipose tissue [14,15,16], menstrual blood [17], peripheral blood [18], salivary glands [19], skin and foreskin [20], and dental tissues [5,21].

However, owing to their neural crest origin, hDPSCs are considered to have a heightened propensity for neuronal differentiation compared to other MSCs [15,22], expressing neural/stem cell markers such as βIII-tubulin, GFAP, and Nestin, even in an undifferentiated state [23].

Dental pulp, including its stem cells (DPSCs), origin ates from the neural crest. This tissue forms the dental papilla, which is a direct precursor of the dental pulp. This neural crest origin is why DPSCs retain a strong innate potential to differentiate into neuronal and other cell types [24].

Several studies have demonstrated that among the eight types of mesenchymal cells that can be obtained from a tooth, hDPSCs have the greatest potential for neurogenic differentiation [25]. As previously mentioned, DPSC have common embryological origin with neural crest cells. In addition, they have high proliferation rates and can expand in vitro within a few weeks. They can be obtained in a less traumatic and simpler way than other mesenchymal stem cells, which is why they have been considered ideal candidates for neuronal regeneration therapy. They could be used for cell therapy in pathologies such as cerebrovascular, ischemia, traumatic brain injury, and neurodegenerative diseases, such as Parkinson’s, Huntington’s, and Alzheimer’s diseases [25,26]. Although MSC-based therapy holds great potential, further research is required to ensure its safety and efficacy in humans.

Similar to MSCs, hDPSCs can be differentiated into different cell types, including odontoblasts, osteoblasts, chondrocytes, cardiomyocytes, neuronal cells, adipocytes, epithelial cells, melanocytic cells, and β-pancreatic cells, in different inductive media [27], even though hDPSCs express markers of neural stem cells such as GFAP, HNK-1, Nestin, P75NTR, S-100, and βIII-tubulin [6,23].

This inherent neurogenic potential has been demonstrated in vitro, with studies showing that under specific culture conditions, hDPSCs can upregulate mature neuronal markers and exhibit neuron-like electrophysiological activity [28,29]. In animal models, transplanted hDPSCs integrate into host neural tissue, express neuronal markers, and promote functional recovery after neurological injury [26,30].

Despite this promise, a significant barrier to clinical translation and reproducible research is the reliance on culture systems supplemented with fetal bovine serum (FBS). FBS introduces batch-to-batch variability, risks of xenogeneic immune reactions, and an undefined cocktail of factors that obscure specific mechanistic insights [31]. Therefore, the development of fully defined, xeno-free culture conditions is a critical prerequisite for the advancement of safe and effective cell-based therapies [32,33,34]. Growth factors such as GDNF and BDNF are known to support neuronal survival and differentiation [35]. Their specific efficacy in driving the terminal neuronal fate lineage of hDPSCs within a fully chemically defined system remains inadequately explored, particularly using functional electrophysiological benchmarks.

Therefore, the hypothesis of this study is that GDNF is a critical driver of neuronal fate lineage in hDPSCs and that its effects can be optimally elicited and evaluated in a serum-free, chemically defined medium. This study aimed to develop a novel, fully defined protocol to drive the neuronal fate lineage of hDPSCs from a Mexican donor, rigorously assessing differentiation using molecular markers and whole-cell patch-clamp electrophysiology to evaluate functional neuronal properties.

### Chemically Defined Versus Serum-Supplemented Media for Neural Stem Cell Differentiation

The choice between a chemically defined medium and a complete medium supplemented with fetal bovine serum (FBS) represents a critical methodological decision in neural stem cell differentiation protocols, with profound implications for reproducibility and clinical translation in regenerative medicine. [36] demonstrated that mouse embryonic stem cells could undergo neural differentiation in a simplified monolayer culture by initially culturing cells in serum-containing DMEM for two days, followed by transfer to serum-free DMEM/F12 medium supplemented with insulin, transferrin, selenium, and fibronectin (ITSF), which successfully drove the majority of ES cells toward neural lineage without requiring embryoid body formation or retinoic acid treatment [36]. In contrast, another study [37] that isolated and expanded multipotent CNS stem cells from the adult mammalian spinal cord and ventricular neuroaxis entirely in serum-free medium containing epidermal growth factor (EGF) and basic fibroblast growth factor (bFGF) demonstrated that these defined mitogens could maintain stem cell proliferation and multipotency [37]. The fundamental difference between these approaches lies in the presence of undefined, batch-variable components in FBS, as opposed to the precise control afforded by chemically defined supplements. While serum-containing media offer simplicity and can support spontaneous differentiation, they introduce significant variability in gene expression and differentiation outcomes between batches, complicating experimental reproducibility and standardization across laboratories [38]. Conversely, chemically defined serum-free media, such as those supplemented with specific growth factors (EGF, bFGF) or defined supplements (Insulin, Transferrin, and Selenium (ITS)), enable selective expansion of neural stem cell populations, reproducible mitogenic control, and predictable responses to factor withdrawal for directed differentiation [39]. This reproducibility is essential for regenerative medicine applications, where regulatory approval requires standardized, well-characterized cell products that are free from xenogeneic contaminants. Serum-free differentiation protocols have demonstrated the generation of functional neuronal phenotypes that survive transplantation in preclinical models, supporting their translational potential [40]. However, successful clinical translation requires careful optimization of growth factor timing, lineage specification signals, and complete removal of exogenous mitogens before transplantation to ensure predictable differentiation, prevent unwanted proliferation, and maximize safety [39,41]. The evolution from serum-dependent to fully defined culture systems, as exemplified by the progression from the Weiss serum-free expansion protocol to the Pachernik hybrid approach, reflects the field’s movement toward more controlled, reproducible, and clinically viable neural stem cell differentiation strategies that are essential for advancing regenerative medicine therapies for neurological disorders. In the present work, we sought to use a fully chemically defined medium to differentiate hDPSCs, based on the above-mentioned defined medium used previously to maintain and differentiate murine stem cells [36,37].

In summary, patient-derived mesenchymal cells from various stem cell niches, such as dental tissue, can be used to model degenerative diseases, providing a better understanding of the molecular processes involved in neurodegenerative diseases and enabling drug design and testing in the future. Therefore, we propose an improved differentiation protocol for hDPSCs from a Mexican donor to obtain neuronal cells by inducing differentiation with neurotrophic factors in a serum-free, fully chemically defined medium.

## 2. Materials and Methods

### 2.1. Isolation and Primary Culture of hDPSCs

This study was conducted in accordance with the Declaration of Helsinki and approved by the Research and Ethics Committees of the Research Division of the Faculty ol of Medicine, UNAM (Project No. FM/DI/023/2017).

An informed consent letter was signed prior to hDPSCs isolation. hDPSCs were obtained from the first molar of a 46-year-old apparently healthy Mexican female volunteer (without cognitive impairment) through routine tooth extraction with informed consent. This study was approved by the Research and Ethics Committee of the Faculty of Medicine at the National Autonomous University of Mexico (UNAM).

The dental pulp was transferred and kept in a saline solution at 4 °C before digestion to preserve the integrity of the sample. The teeth were transversely sliced using a dental micromotor. The dental pulp was isolated using number 5 sterile forceps and immersed in a digestion solution (3 mg/mL type I collagenase/dispase in DMEM-F12). After incubation for 45 min at 37 °C, shaking every 10 min, the enzymes were inactivated with culture medium (DMEM-F12 plus 10% FBS) and resuspended in fresh medium. hDPSCs were seeded in a 25 cm^2^ bottle with complete culture medium (DMEM-F12 with 10% inactivated fetal bovine serum, 2 mM L-glutamine, 100 U/mL penicillin, and 100 mg/mL streptomycin), and the bottle was placed in an incubator at 37 °C and 5% CO_2_. hDPSCs were expanded to 70% confluence. Cell passages were performed using 0.05% trypsin-EDTA. Cryopreservation was performed in complete DMEM-F12 medium plus 10% FBS at −70 °C. All culture reagents were purchased from Invitrogen (Carlsbad, CA, USA). A fully defined medium was used for hDPSCs neural differentiation.

### 2.2. Phenotype Characterization by FACS

hDPSCS were characterized based on their expression of MSCs markers, morphology, and differentiation potential. Phenotypic markers evaluated: CD90 PE-Cy5, CD73 PE-Cy7, CD13 APC, HLA-ABC FITC, HLA-DR PE, CD45 PE, CD34 APC, CD31 FITC, CD14 FITC (Biosciences, San Diego, CA, USA) and CD105 PE (Biolegend, San Diego, CA, USA). These were used for immunophenotypic characterizations and analyzed by FACS Canto II flow cytometer (Biosciences, San Diego, CA, USA), and the data were analyzed using FlowJo 10 software (FlowJo LLC, Ashland, OR, USA). Adipogenic differentiation was induced for 21 days in the presence of Dexamethasone, 3-Isobutyl-1-methylxanthine, insulin, and indomethacin. Adipogenic differentiation was determined by visualizing the presence of Oil Red O-stained (Sigma-Aldrich, St. Louis, MO, USA) lipid vacuoles. Osteogenic differentiation was induced for 21 days using dexamethasone, β-glycerophosphate, ascorbic acid, and 2-Phospho-L-ascorbic acid. Osteogenic differentiation was assessed using alkaline phosphatase staining (FAST BCIP/NBT, MilliporeSigma, Burlington, MA, USA). Chondrogenic differentiation was induced for 21 days with Dexamethasone, Sodium pyruvate, Proline, 2-Phospho-L-ascorbic acid, ITS, and TGF-β, and was evaluated using Alcian blue dye (MilliporeSigma, Burlington, MA, USA) [42].

### 2.3. Neural Differentiation of hDPSCs

hDPSCs were first grown in complete medium (DMEM-F12 plus 10% inactivated FBS) in a T25 culture bottle until reaching 70–80% confluence (approximately 1,000,000 cells per 25 cm^2^). Subsequently, the cells were subcultured at a density of 200,000 cells per 25 cm^2^ bottle in DMEM-F12 complete culture medium with 10% FBS for 24 h. Next, the medium was changed to a fully chemically defined medium: Insulin, Transferrin, and Selenium (ITS) 1% medium based on previously reported culture media [36,37], containing DMEM-F12, 2 mM L-glutamine, 100 U/mL penicillin, 100 mg/mL streptomycin, 0.1 M putrescine, 10 µM progesterone, and 1% insulin–transferrin–selenium (ITS) mixture supplemented with 5 ng/mL bFGF and 5 ng/mL EGF for three days. This process is called a transition. After three days, the cells were subcultured, and the medium was changed to a differentiation medium consisting of ITS 1% medium plus combinations of neurotrophic factors as indicated: (a) ITS 1% medium alone (without growth factors), (b) ITS 1% medium + 20 ng/mL BDNF, (c) ITS 1% medium + 20 ng/mL GDNF, and (d) ITS 1% medium + 20 ng/mL BDNF + 20 ng/mL GDNF. The cells were maintained in these media for 5 or 35 days, with medium changes every third day, and subsequently evaluated by immunodetection, endpoint PCR, and electrophysiology.

### 2.4. RNA Extraction

RNA was extracted from differentiated hDPSCs using the Zymo Research Quick-RNA ™ MicroPrep (Plus) KIT (Cat. No. R1057, Zymo Research, Irvine, CA, USA). Adherent cells were removed from the medium, and 100 µL of lysis solution was added. The lysates were collected and centrifuged at 12,000 rpm for 1 min. The supernatant was transferred to an RNase-free tube; one volume of 100% ethanol was added, mixed, and transferred to a Zymo-Spin column with a collecting tube and centrifuged for 30 s at 12,000 rpm. The liquid was discarded from the collector, and the column was pre-washed with 400 μL of RNA wash solution and centrifuged for 30 s at 12,000 rpm. The liquid in the collector was discarded, and 40 μL of DNase I (DNase I2 5 μL + DNA digestion solution 35 μL) was added to the column, incubated at 25 °C for 15 min, and centrifuged for 30 s at 12,000 rpm; the liquid was discarded from the collection tube, 400 μL of Prep RNA solution was added to the column and centrifuged for 30 s at 12,000 rpm, and the contents of the collection tube were dried. Next, 700 µL of RNA wash solution was added to the column, centrifuged for 30 s at 12,000 rpm, and the collecting tube was discarded. Next, 400 μL of RNA wash solution was added and centrifuged for two min at 12,000 rpm until the wash solution was completely removed. The column was transferred to an RNase-free tube, and 15 µL of RNase-free water was placed directly on the column and centrifuged for 30 s. The eluted RNA was stored at −70 °C.

### 2.5. Reverse-Transcription

The BIOLINE Tetro reverse transcriptase system (Cat. No. BIO-65050, Bioline, Meridian Bioscience, Memphis, TN, USA) was used. The samples were mixed according to the following protocol.

Total RNA 5 μg μL, Ramdom hexamers (40 μM) 1 uL, 10 mM dNTPs mixture 1 uL, 5× Buffer RT, 4 uL, RiboSafe Rnase Inhibitor 1 uL, Tetro Reverse Transcriptase (200 u/μL) 1 uL, Treated water DEPC up to 20 μL.

The samples were incubated for 10 min at 25 °C, followed by incubation at 45 °C for 30 min. The reaction was stopped by incubation at 85 °C for 5 min, and stored at −20 °C or used in PCR.

### 2.6. Endpoint PCR

Amplification of hDPSCs characterization and neuronal differentiation genes was performed using the BIOLINE MyTaq ™ DNA Polymerase PCR KIT (Cat. No. BIO-21105, Bioline, Meridian Bioscience, Memphis, TN, USA), under the following reaction conditions (Table 1):

A total of 5× Taq Reaction Buffer BIOLINE 4 μL, cDNA, forward primer (0.1 μg/μL, reverse primer (0.1 μg/μL) (Table 2) 1 uL, DNA polymerase BIOLINE 0.3 μL, sterile H_2_O molecular biology grade (the volume needed to reach the final volume of 20 μL) for 20 μL, total for 1 reaction 20 μL

Amplification cycle: Initial denaturation at 95 °C for 5 min, denaturation at 95 °C for 45 s, alignment for 45 s, extension at 72 °C for 45 s, for 40 cycles, and a final extension at 72 °C for 7 min.

Reactions were visualized using a transilluminator with ethidium bromide-stained agarose gels.

### 2.7. Protein Extraction

The culture medium was removed, and the cells were washed with glucose-buffered saline, GBS (5.4 mM KCl, 138 mM NaCl, 22 mM glucose, 2 mM Na-KPO4, pH 7.2) at 4 °C or with PBS (phosphate-buffered saline, pH 7.4). Cold RIPA 1× buffer (1× PBS, 1% (*w*/*v*) NP-40 (Thermo Fisher Scientific, Waltham, MA, USA), 0.1% (*w*/*v*) SDS, and 0.5% (*w*/*v*) deoxycholate containing protease/phosphatase inhibitors (1 mM AEBSF (Gold Biotechnology, St. Louis, MO, USA), 20 μg/mL of leupeptin and pepstatin (US Biochemicals, Cleveland, OH, USA), 10 mM NaF, 1 mM Na orthovanadate, and 20 mM β-glycerophosphate (Sigma-Aldrich) were added directly to the plate (100–150 µL per well for a 6-well plate, or 20 µL for a T-25 box). The cells were incubated for 5 min on ice and shaken manually to completely detach the monolayer. The lysate was transferred to a 1.5 mL tube and frozen at −20 °C for 16 h. The next day, the sample was thawed and resuspended before centrifuging at 14,000 rpm for 10 min at 4 °C. The supernatant was transferred to a clean tube, and an aliquot was taken to determine the protein content using the bicinchoninic acid (BCA) assay (Cat. No. 23227 Pierce BCA Protein Assay Kit, Thermo Fisher Scientific, Waltham, MA, USA).

### 2.8. Western Blot

Cells were lysed in RIPA 1× buffer (4× PBS, 0.4% SDS, 4% NP-40, 2% sodium deoxycholate, 1000× AEBSF, 10 mg/mL aprotinin, 10 mg/mL leupeptin, 2 mg/mL pepstatin, 500 mM benzamidine, 50×, NaF at 1 mM, O-vanadate at 250 mM O-vanadate, 0.5 M EDTA pH 8, 0.5 M EGTA pH 8). Each 10 µg protein sample was subjected to SDS-PAGE (SDS-polyacrylamide gels with a concentration of 8 to 10%), and the separated proteins were transferred to a 0.22 μm nitrocellulose membrane. The membranes were incubated with primary antibodies (Table 3), followed by secondary antibodies according to the species of the primary antibody, and they were visualized using chemiluminescence (ECL). Images were acquired using the MicroChemi image system (DNR). The densitometry of the protein bands was analyzed using Fiji ImageJ software (version 1.54t).

### 2.9. Immunocytofluorescence

Cells were fixed in 4% (*m*/*v*) paraformaldehyde in phosphate buffer (PBS) for 15 min and washed 2 times with GBS for 10 min. Fixed cells were permeabilized by incubation in 0.25% (*m*/*v*) Triton X-100 in PBS for 10 min, and non-specific binding was blocked by incubating with 1% (*m*/*v*) bovine serum albumin (*m*/*v*) in PBS for 30 min. Subsequently, the cells were incubated with primary antibody overnight in Triton PBS and 1% BSA, washed, and incubated with secondary fluorescein antibodies Alexa 488 or Alexa 546 (Thermo Fisher Scientific) in PBS plus 1% BSA for 1 h, and nuclei stained with DAPI at 10 μg/mL for 10 min. The coverslips were mounted with Fluorogel (Electron Microscopy Sciences, Hatfield, PA, USA) and allowed to dry overnight.

### 2.10. Confocal Mycroscopy

For hDPSC immunophenotypic characterization, the expression of CD105, CD73, Ki67, Nestin, βIII-tubulin (Tuj-1), and GFAP were analyzed. In the differentiation assays, cells from each experimental group, according to the corresponding treatment condition (ITS 1% alone, BDNF, GDNF, or BDNF + GDNF), were evaluated for the expression of Ki67, Nestin, βIII-tubulin (Tuj-1), and GFAP. In both the characterization and differentiation experiments, a minimum of 5 fields per condition were analyzed across 3 independent experiments, using randomly selected cells. Images were captured using a Leica TCS SP8 confocal microscope, and the z-sections were processed and analyzed using FIJI ImageJ. All images presented here are maximum-intensity projections of the z-sections, unless otherwise noted.

### 2.11. Electrophysiology

Patch-clamp measurements were recorded using an Axopatch 200 A amplifier (Axon Instruments, Foster City, CA, USA). The data were acquired at a sampling rate of 20 kHz and low-pass filtered (4 kHz). To measure the passive properties of the membrane, depolarization pulses of 10 mV for 200 ms were applied with a holding potential of −80 mV. Data was analyzed using pCLAMP 8.0 (Axon Instruments, Foster City, CA, USA). The pipettes had a resistance of–4–5 MΩ. The standard pipette solution (pH 7.2) contained (in mM): 2 MgCl_2_, 125 KCl, 10 HEPES, 1 CaCl_2_, 10 EGTA, 2 Mg-ATP, and 2 Na2ATP. The bath solution (pH 7.4) contained (in mM): 2 CaCl_2_, 145 NaCl, 2 MgCl_2_, 10 HEPES, 1.5 KCl, and 10 glucose.

### 2.12. Statistics

Statistical analyses were conducted using GraphPad Prism 7.0 (GraphPad Software, La Jolla, CA, USA) to evaluate the differences in expression among the different treatments and conditions. Data are presented as the mean ± standard deviation. For analyses involving multiple groups, one-way ANOVA with post hoc Fischer’s, Tukey’s, or Bonferroni’s test (as appropriate) was used. For all other comparisons (including inter-group comparisons), Student’s *t*-test was used. For all purposes, *p* ≤ 0.05 was considered statistically significant.

## 3. Results

### 3.1. Establishment and Characterization of Mesenchymal Stem Cells from the First Molar Dental Pulp

**Figure 1:**
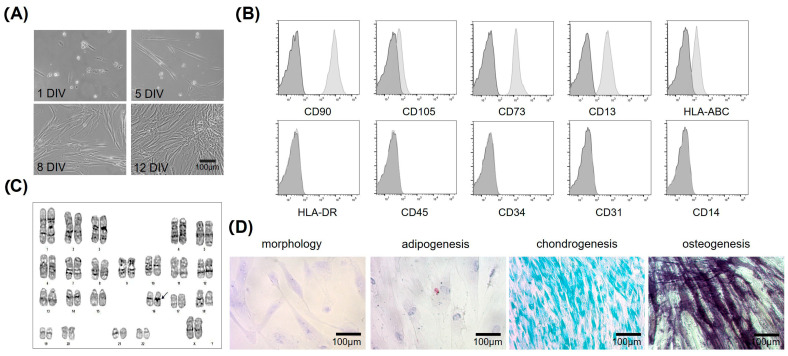
Comprehensive characterization of mesenchymal stem cells derived from human dental pulp of a first molar: morphological analysis, cell surface marker expression, and differentiation potential (**A** ) hDPSCs grown in medium containing 10% FBS showed adherence to the plastic culture bottle and fibroblast-like cell morphology at 1, 5, 8, and 12 days in vitro (DIV). The arrangement of hDPSCs at confluence was organized and aligned at 8 DIV. The cultures were at passage 3. Scale bar = 100 μm. (**B**) Cell surface marker analysis of MSCs from dental pulp (expression of cell markers was determined by flow cytometry) showed positive expression of CD90, CD105, CD73, and CD13 and negative expression of HLA-DR, CD45, CD34, and CD31. MSCs were incubated with antibodies and analyzed using flow cytometry. Autofluorescence (dark gray histograms) and positive populations (light gray histograms) are shown. (**C**) Karyotype of a 46-year-old female was observed, with a normal chromosome number (46) and an increase in normal centromeric heterochromatin on chromosome 16 (16qh+). (**D**) In vitro lineage differentiation potential of MSCs from dental pulp, morphology was stained with Wright staining; adipogenic differentiation was indicated by lipid droplets stained with Oil Red O; Chondroblastic differentiation was indicated by chondrogenic matrix, as indicated by Alcian blue staining; osteoblastic differentiation was confirmed by alkaline phosphatase staining. Scale bar = 100 μm.

In the present study, a single subject was selected for the study, focusing on investigating and optimizing the neural differentiation protocol in the first molar of a 46-year-old female Mexican donor. Our cultures have shown that mesenchymal stem cells derived from dental pulp (hDPSCs) exhibit self-renewal capacity, allowing them to proliferate while maintaining an undifferentiated state. The major advantage of hDPSCs is that they are not genetically modified and possess multipotent differentiation capacity. hDPSCs cultured showed fibroblastic-like morphology, with large cytoplasm, and presented partially homogenous orientation at high culture densities and proliferation through the in vitro passages (we cultured these hDPSCs for no more than 10 in vitro passages to avoid cell senescence or loss of proliferation capacity) (Figure 1A). Our cultured hDPSCs met the requirements to be considered MSCs according to the “Mesenchymal and Tissue Stem Cell Committee” of the International Society of Cell Therapy, which proposed the minimum criteria to define human mesenchymal cells [12]: (1) ability to adhere to plastic; (2) the expression pattern of the surface markers known as “Cluster of Differentiation,” being positive for CD105 (endoglin), CD73 (5′-nucleotidase) and CD13 (alanyl-aminopeptidase), and negative for CD45, CD34, CD14 or CD11b, CD79 or CD19 and surface molecules such as HLA-DR; and (3) the potential to differentiate in vitro to osteoblasts, adipocytes and chondroblasts. The expression of CD90 PE-Cy5, CD73 PE-Cy7, CD13 APC, HLA-ABC FITC, HLA-DR PE, CD45 PE, CD34 APC, CD31 FITC, CD14 FITC, and CD105 PE in hDPSCs cultures was assessed for immunophenotypic characterization and analyzed using a FACS Canto II flow cytometer (Figure 1B). Furthermore, a comprehensive evaluation of hDPSCs marker expression was performed using flow cytometry (Figure S1) and found to correlate with the mesenchymal phenotype. A normal karyotype of a 46-year-old female was confirmed with a chromosome number of 46, with an increase in normal centromeric heterochromatin on chromosome 16 (16qh +) obtained using the G-band karyotype technique (Figure 1C). This finding is not relevant to the present investigation, since 16qh+ is a heteromorphic variant without direct phenotypic consequences in many carriers [43,44].

Mesenchymal cells are considered multipotent progenitor cells capable of responding to specific differentiation stimuli. To confirm this, an in vitro differentiation assay was performed. Adipogenic differentiation was induced for 21 days in the presence of Dexamethasone, 3 Isobutyl-1-methylxanthine, insulin, and indomethacin. Adipogenic differentiation was confirmed by Oil Red O staining of lipid vacuoles (Figure 1D). Osteogenic differentiation was induced for 21 days using dexamethasone, β-glycerophosphate, ascorbic acid, and 2-Phospho-L-ascorbic acid. Osteogenic differentiation was confirmed via alkaline phosphatase staining (Figure 1D). Chondrogenic differentiation was induced for 21 days with Dexamethasone, Sodium pyruvate, Proline, 2-Phospho-L-ascorbic acid, ITS, and TGF-β and was confirmed with Alcian blue dye (Figure 1D). These phenotypic changes were observed under a light microscope and confirmed that the hDPSCs used in our study retained the characteristic plasticity of MCS by differentiating into chondroblasts, osteoblasts, and adipocytes with the inducing media, in addition to the expression of corresponding phenotypic markers and their adherence to plastic (Figure 1).

Next, the mesenchymal phenotype of hDPSCs was corroborated by the expression of the markers CD10,5 and CD73 by immunofluorescence5 (Endoglin), which associated with the Transforming Growth Factor Receptor β (TGFβR), while CD73 is an enzyme (5′-nucleotidase, 5′-NT) encoded by the NT5E gene [45], used as a marker of differentiation of lymphocytes and MSCs.

**Figure 2:**
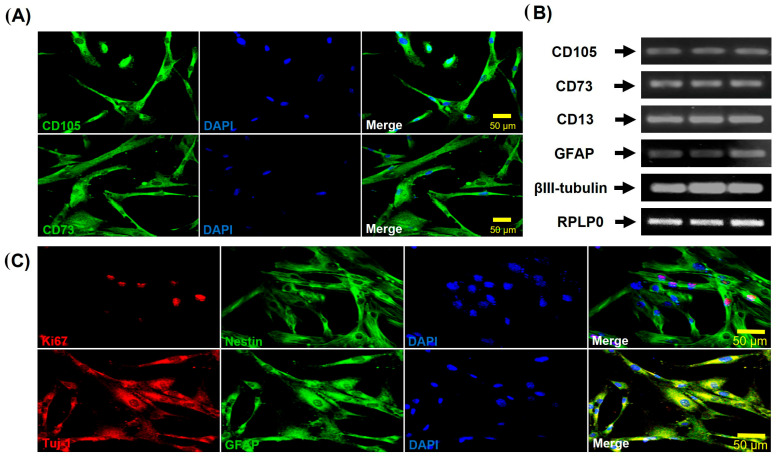
Characterization of specific markers expressed by MSCs of the dental pulp when cultured in 10% FBS. (**A**) Immunofluorescence analysis showed positive expression of mesenchymal-specific markers CD73 and CD105 observed in green, nuclei were counterstained with DAPI (blue). hDPSCs were in passage 5. Scale bar = 50 μm. (**B**) RT-PCR expression of mesenchymal (CD105, CD13, and CD73) and neural (GFAP and βIII-Tubulin (Tuj-1))-specific markers in MSCs. RPLP0 was used as the housekeeping gene control. (**C**) Immunofluorescence analysis showed positive expression of neural markers. Dental pulp cells are proliferative, as indicated by the expression of the nuclear marker Ki67 (red), and express early neural markers such as Nestin and GFAP (green), and βIII-Tubulin (Tuj-1) (red). DAPI was used to stain the nuclei. hDPSCs were cultured at passage 5. Scale bar = 50 µm. hDPSCs were grown in a medium containing 10% FBS.

As previously mentioned, hDPSCs are derived from cranial neural crest cells during embryonic development [6] and they maintain the expression of markers for neural stem cells, such as GFAP, HNK-1, Nestin, P75, S-100, and βIII Tubulin. Evaluation was performed using immunofluorescence and end-point RT-PCR for the expression of relevant markers, such as Ki67 (proliferation) and neural markers (Nestin, GFAP, and βIII Tubulin). These results showed that hDPSCs have neural characteristics when cultured in the growing medium (with 10% FBS) before being induced to differentiate in the chemically defined medium (ITS 1% plus growth factors) (Figure 2).

**Figure 3:**
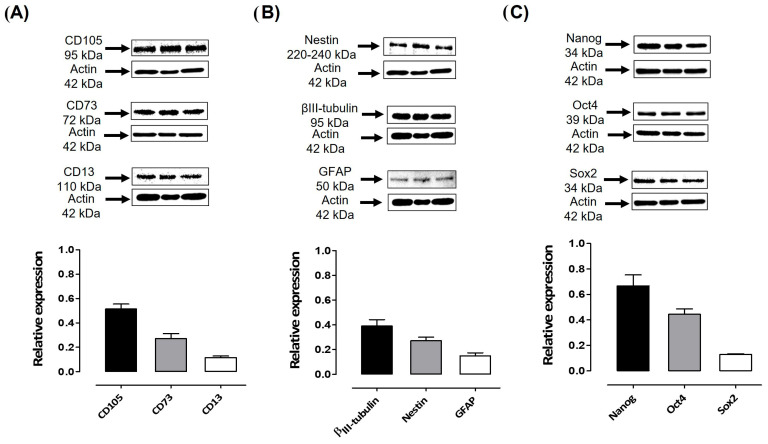
Expression of mesenchymal, neural, and pluripotent markers in dental pulp stem cells cultured in the presence of 10% FBS. (**A**) Immunoblots of hDPSCs cultured in medium containing 10% FBS showed the expression of mesenchymal markers CD105 (95 kDa), CD73 (72 kDa), and CD13 (110 kDa). (**B**) Immunoblots of hDPSCs cultured in medium containing 10% FBS showed positive expression of neural markers Nestin (220–240 kDa), βIII-Tubulin (Tuj-1) (50–55 kDa), and GFAP (50 kDa). (**C**) Immunoblots of hDPSCs cultured in medium containing 10% FBS showed the expression of Nanog (35 kDa), Oct4 (43–50 kDa), and Sox2 (34 kDa). β-actin (42 kDa) was used as the loading control. Graphs depict the densitometric analysis of the expression of the evaluated pluripotent and neural markers relative to β-actin levels.

In addition to expressing mesenchymal (CD105, CD73, and CD13) (Figure 3A) and neural (Nestin, GFAP, and βIII Tubulin) markers (Figure 3B), hDPSCs also express pluripotency markers such as Oct4, Nanog, Sox-2, SSEA-4, and TRA-160 [46]. The expression levels of Nanog, Oct4, and Sox-2 were assessed using Western blotting (Figure 3C). These data confirm the mesenchymal identity and differentiation capacity of the hDPSCs used in this study. hDPSCs express mesenchymal, neural, and pluripotency markers. Although these cells are multipotent, it has been reported that hDPSCS mesenchymal stem cells express pluripotency markers, but not at a fully functional level [46,47]. Furthermore, Oct-4 and Nanog, markers of pluripotent cells, are downregulated following the differentiation of these cells [48].

### 3.2. Transition and Differentiation of hDPSCs

**Figure 4:**
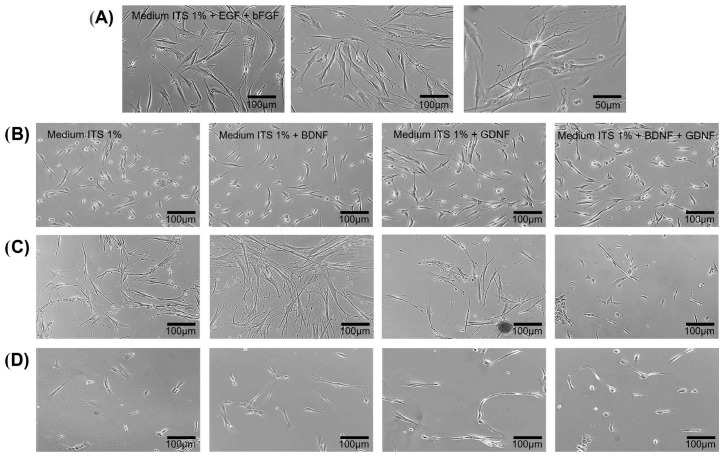
In vitro differentiation of hDPSCs cultured in a chemically defined medium (ITS 1% with or without growth factors). Phase-contrast photomicrographs of hDPSCs cultures: (**A**) hDPSCs cultured in transition medium (ITS 1%, 5 ng/mL of EGF and 5 ng/mL of bFGF), the cells show morphological changes with neuritic-type, decreased cytoplasmic area, and radial morphology, three days in vitro (DIV). Scale bars = 50 μm and 100 μm, respectively. (**B**–**D**) hDPSCs were cultured in the differentiation Medium ITS different stimuli (at different time points): Medium ITS at 1%, Medium ITS at 1% + BDNF, Medium ITS at 1% + GDNF, Medium ITS at 1% + BDNF + GDNF. B (5 days) C (24 days) D (35 days).

Numerous studies have been conducted to establish effective culture strategies that promote the differentiation of mesenchymal cells from the dental pulp into the neuronal lineage. Since FBS contains an unknown “undefined mixture” of growth factors and hormones, we switched to a chemically defined medium (1% ITS) to induce differentiation under controlled culture conditions. However, removing FBS from the culture medium could cause cell death; therefore, it was necessary to gradually transition to a medium without FBS. Accordingly first, we cultivated the hDPSCs in medium containing 10% of FBS, then we changed the medium to a fully chemically defined culture medium (Insulin Transferrin Selenium 1%) plus 5 ng/mL of bFGF and 5 ng/mL of EGF, to induce adapting the cells to growth without FBS; in this condition, changes in the morphology of the hDPSCs were observed, which is known as a transition process. The morphological changes included cells with neurite-like extensions, reduced cytoplasmic area, and radial forms (Figure 4A). After this process, the cells were subcultured to induce their differentiation under different conditions with the neurotrophic factors BDNF and/or GDNF (Figure 4B–D).

The differentiation of hDPSCs was induced under different culture conditions: Medium ITS at 1% (alone, without growth factors), Medium ITS at 1% + BDNF, Medium ITS at 1% + GDNF, and Medium ITS at 1% + BDNF + GDNF, and the changes in morphology were followed for 5, 24, and 35 days. This study showed that BDNF induced bipolar morphology and neurite-like extensions at 5 DIV (Figure 4B), while GDNF induced, to a lesser extent, fine neuritic growth and the development of bipolar cells with the presence of fibroblastic-like forms (Figure 4C). Neuroblast- and fibroblast-like morphologies were observed under a combination of trophic factors (BDNF + GDNF) (Figure 4B–D). ITS 1% alone induced neural forms to a lesser extent, and the presence of fibroblastic-like morphology.

**Figure 5:**
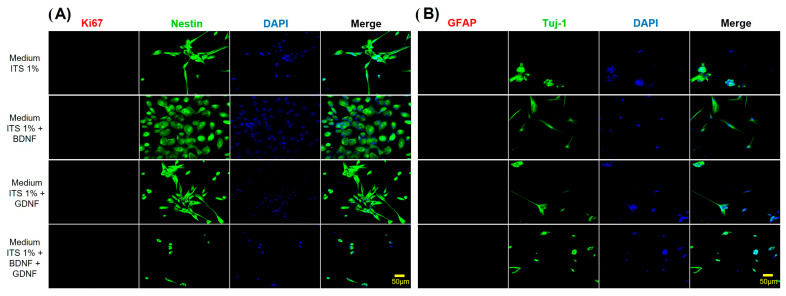
In vitro differentiation of hDPSCs into neuronal-like cells cultured in a chemically defined medium (ITS 1% with or without growth factors). (**A**) Immunofluorescence analysis showed positive expression of the neural-specific marker Nestin and loss of expression of the proliferative marker (Ki67). DAPI was used to stain the nuclei. Cells were cultured in differentiation medium for 5 days, as indicated on the left side of the figure. (**B**) Immunofluorescence analysis showed positive expression of the neuronal-specific marker βIII-tubulin (Tuj-1). The nuclei were counterstained with DAPI (blue) after five days of treatment. Scale bar = 50 μm.

**Figure 6:**
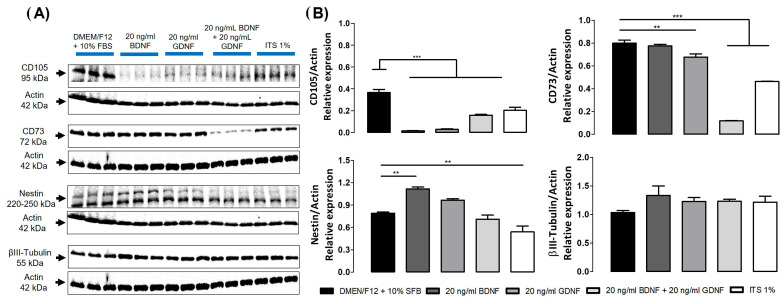
Immunoblot analysis of hDPSC markers under differentiation (ITS 1% with or without growth factors) and non-differentiation (10% FBS) conditions after 5 days of culture. (**A**) Immunoblot of the expression of mesenchymal markers (CD105 and CD73) and neural markers (Nestin and βIII-Tubulin (Tuj-1)). β-Actin (42 kDa) was used as the loading control for Western blotting. (**B**) Densitometric analysis, graphs depict the relative density of mesenchymal and neural markers to β-actin. Statistical significance was assessed using one-way ANOVA (* *p* ≤ 0.05, ** *p* ≤ 0.01, and *** *p* ≤ 0.001).

The expression of markers of hDPSCs subjected to differentiation during 5 DIV with neurotrophic factors and basal differentiation medium (chemically defined medium) (ITS 1%) was evaluated, and it was observed that during the differentiation process, the expression of Ki67 (proliferation marker) was lost, and the presence of the glial marker GFAP was not detectable (Figure 5). However, hDPSCs under these conditions maintained the expression of neural markers such as Nestin and βIII Tubulin (Tuj-1); nevertheless, the expression of mesenchymal markers, such as CD105 and CD73, decreased (Figure 6).

**Figure 7:**
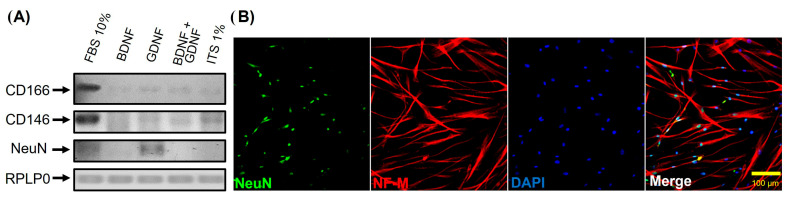
hDPSCs cultured in differentiation medium (ITS 1% with or without growth factors) for 35 DIV expressed mature neuronal differentiation markers. (**A**) End-point RT-PCR evaluation of the expression of CD166, CD146, and NeuN in hDPSCs under different culture conditions for 35 days (DIV): FBS 10%, ITS 1% + BDNF, ITS 1% + GDNF, ITS 1% + BDNF + GDNF, and ITS 1% alone. RPLP0 was used as internal control. (**B**) Immunofluorescence analysis showed positive NeuN and Neurofilament (NF-M) expression at 35 days in vitro in hDPSCs cultured with medium ITS 1% + GDNF. Scale bar = 100 μm. The nuclei were counterstained with DAPI (blue).

Culture of hDPSCs differentiated for 35 DIV showed the presence of cells with predominant neuronal morphology in the defined medium (ITS 1%) plus BDNF or GDNF (Figure 4). To confirm this, end-point PCR of the mature neuronal marker NeuN and the mesenchymal markers CD166 and CD146 were performed, and an evident increase in NeuN expression with a decrease in CD166 and CD146 expression was observed (Figure 7).

hDPSCs cultured in chemically defined medium plus GDNF (ITS 1% + GDNF for 15 days) showed a more differentiated phenotype, expressing NeuN. Passive membrane properties refer to the intrinsic electrical characteristics of a cell membrane that determine its response to subthreshold electrical signals (i.e., signals that do not trigger action potentials). These properties govern the passive spread of electrical signals, such as electrotonic potentials, across the membrane. The key passive membrane properties include membrane capacitance (Cₘ) and input resistance (Rᵢₙ). We measured these two properties and the resting potential.

**Figure 8:**
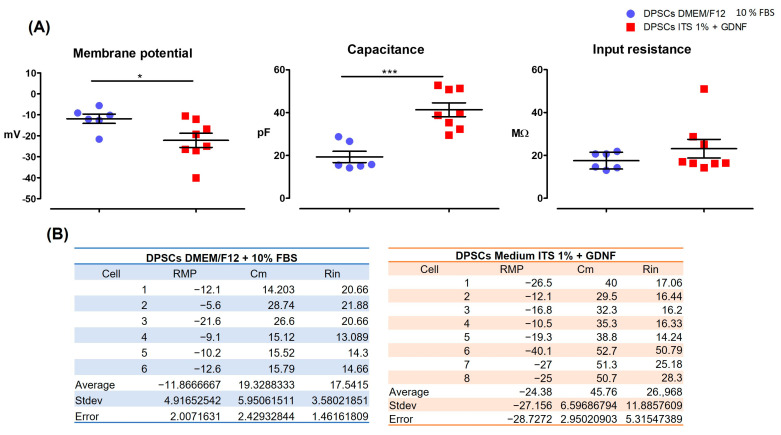
Passive electrical properties of hDPSCs cultured in differentiation (ITS 1% + GDNF) and nondifferentiation media (with 10% FBS) for 15 DIV. hDPSCs were cultured for 15 days in vitro (DIV) in either (**A**) complete medium (DMEM/F12 + 10% FBS, blue) or (**B**) a defined differentiation medium (ITS 1% + 20 ng/mL GDNF, red). Cells cultured in the defined medium (B) exhibited a more negative resting membrane potential (RMP), increased membrane capacitance (Cm), and higher input resistance (Rin), indicating a more mature functional phenotype. Data are presented as mean ± SD. *p* ≤ 0.05 (* *p* ≤ 0.05, ** *p* ≤ 0.01, and *** *p* ≤ 0.001)., unpaired Student’s *t*-test, n = 5 per group.

Moreover, we found that hDPSCs cultured for 15 days in the differentiation medium (defined medium ITS 1% + GDNF) had a more negative RMP, increased capacitance, and increased input resistance compared to hDPSCs cultured in complete medium with 10% FBS) (Figure 8). All of these changes point to a partially more mature phenotype and a more functional stage of these cells when cultured in the differentiation medium (defined medium ITS 1% + GDNF), although the resting membrane potential remains in the corresponding values of mesenchymal stem cells, such as the hDPSCs phenotype (−20 mV to −40 mV) [49,50].

## 4. Discussion

The limited innate regenerative capacity of the mammalian central nervous system (CNS) has motivated the exploration of alternative non-neural stem cell sources for neuroregenerative therapies. Mesenchymal stem cells (MSCs) derived from various tissues, including human dental pulp, have emerged as promising candidates for regenerative medicine due to their accessibility and multipotency. However, owing to their neural crest origin [6], human dental pulp stem cells (hDPSCs) exhibit a differentiation potential that extends beyond mesenchymal lineages to include a neural progenitor and neuronal phenotypes, as evidenced by the expression of mature neuronal markers [51]. Despite this ability, the definitive capacity of hDPSCs to give rise to functional, synaptically integrated neuron-like cells remains a subject of considerable debate within the field [52]. This controversy is largely attributable to the lack of consensus regarding standardized differentiation protocols and unequivocal morphological, molecular, and functional criteria for defining successful neuronal differentiation.

Here, an improved neuronal differentiation protocol for hDPSCs using a chemically defined medium and two growth factors, BDNF and GDNF, is proposed. To our knowledge, this is the first time that a chemically defined medium (ITS) has been used to induce neuronal differentiation of hDPSCs. These findings highlight the potential of hDPSCs to differentiate in vitro using this protocol, with applications in regenerative medicine and neurodegenerative disease research.

In this study, mesenchymal stem cells were isolated from the dental pulp of an apparently healthy Mexican volunteer’s first molar using enzymatic treatment, similar to the protocol reported by Gronthos et al. [5].

An important limitation of the present study is that all the experiments were performed using hDPSCs derived from a single donor. Although single-donor studies have previously been reported as exploratory pilot approaches [5,53,54], this design substantially limits the generalizability of the present findings. These studies, including the present one, are justified as initial exploratory analyses and preliminary results on possible cellular behaviors and responses to specific stimuli, including the differentiation process using a chemically defined medium supplemented with growth factors.

Therefore, our results under the specific conditions tested here, chemically defined medium supplemented with GDNF, were associated with the acquisition of partial neuronal-like features in this hDPSC population, rather than as a broadly validated differentiation protocol for hDPSCs in general. Future studies with multiple independent donors are required to confirm the robustness, consistency, and broader applicability of these observations.

This initial analysis provides a solid foundation that can serve as a starting point for future research with larger sample sizes, which could allow for the generalization of the findings and validation of the results. In summary, the use of n = 1 is a valid strategy for exploratory studies, as it has been used in previous research [47,54], establishing a framework for future studies.

The isolated hDPSCs adhered to the polystyrene culture plate, which is in concordance with MSCs characteristics. Nevertheless, the adhesion properties are not sufficient to classify this cell population as MSCs [55,56], since there are studies in which fibroblasts, macrophages, and some hematopoietic cell lines can adhere to plastic and present morphological characteristics similar to those of MSCs [57]. Furthermore, cell density may also be an influencing factor in the ability of MSCs to adhere to plastic, as low density allows mesenchymal cells to adhere more quickly to plastic [58,59].

The isolated hDPSCs exhibited a fibroblast-like morphology in culture with a defined orientation at high confluence. This fibroblast-like morphology has been reported in mesenchymal stem cells derived from the bone marrow and in various dental tissues, providing a characteristic profile of hDPSCs [5,60,61].

The Mesenchymal and Tissue Stem Cell Committee of the International Society for Cell Therapy proposed the minimum criteria to define human MSCs: (1) ability to adhere to plastic; (2) the expression pattern of the surface markers known as Cluster of Differentiation being positive for CD105, CD73, and CD90, and negative for CD45, CD34, CD14 or CD11b, CD79 or CD19, and surface molecules such as HLA-DR; and (3) the potential to differentiate osteoblasts, adipocytes, and chondroblasts in vitro [11,62]. In the present study, all these criteria for mesenchymal cells were successfully verified in hDPSCs, confirming their phenotype. Although the isolated hDPSCs expressed neural and pluripotency markers, they were multipotent stem cells. Accordingly, it has been reported that hDPSCs mesenchymal stem cells express pluripotency markers, but not at a fully functional level [46,47]. Moreover, we found that Oct-4 and Nanog, markers of pluripotent cells, were downregulated following differentiation, consistent with previous reports [48]. Different methods for the isolation and expansion of hDPSCs have been reported, and their phenotypic characterization has been widely documented [60,63,64,65]. In the present study, the isolated hDPSCs were positive for the following MSCs markers: CD105, CD90, CD73, and CD13. They were negative for hematopoietic markers—HLA-DR, CD45, CD34, CD31, and CD14—as detected by flow cytometry, immunofluorescence, endpoint PCR, and Western blotting. The expression of these markers agrees with previous findings, indicating that these markers are characteristic of MSCs [60,66,67].

When investigating the neural differentiation potential of MSCs, the success of differentiation is based on the expression of neural-associated markers, such as Nestin, βIII-tubulin (Tuj-1), Neurofilament, GFAP, and NeuN, as well as the electrophysiological properties of excitable cells [68,69,70,71]. Neural-associated markers, such as Nestin, βIII-tubulin (Tuj-1), GFAP, and the Ki67 proliferation marker, which are highly expressed in pulp tissue cells, have been identified by immunodetection in human hDPSCs [68,69,70,71]. Accordingly, bone marrow MSCs have been reported to express neural genes when incubated in culture media with a high percentage of serum and epidermal growth factor, leading to Nestin and Neurofilament expression [72]. hDPSCs have also been reported to express low levels of basal neural markers [73]. In addition to the expression of neural markers by hDPSCs, several studies have indicated the production and secretion of neurotrophic factors, such as BDNF, GDNF, and nerve growth factor (NGF), by DPSCs [8,74]. Therefore, our undifferentiated hDPSCs expressed neural progenitor markers (βIII-tubulin (Tuj-1) and Nestin). Interestingly, although both BDNF and GDNF are neurotrophic factors associated with neuronal differentiation, the combined BDNF + GDNF condition did not reproduce the same pattern as GDNF alone. Under our experimental conditions, GDNF treatment was more clearly associated with the expression of later neuronal-like markers, including NeuN and NF-M, whereas the combined treatment appeared to favor mainly earlier neuronal-associated markers. This suggests that the simultaneous presence of both factors does not necessarily result in a synergistic effect in hDPSCs cultured in a chemically defined medium and that GDNF alone may be more favorable for the acquisition of a more advanced neuronal-like phenotype under these culture conditions.

Furthermore, we evaluated passive membrane properties of the hDPSCs cultured under two conditions: in complete medium (DMEM/F12 + 10% FBS) and in defined medium (ITS 1% + GDNF for 15 days) to assess whether the culture conditions in medium ITS 1% + GDNF for 15 days could induce a more mature and functionally committed phenotype. Passive membrane properties refer to the intrinsic electrical characteristics of a cell membrane that determine how it responds to subthreshold electrical signals (i.e., signals that do not trigger action potentials). These properties govern the passive spread of electrical signals such as electrotonic potentials across the membrane. The key passive membrane properties include membrane capacitance (Cₘ) and input resistance (Rᵢₙ). These two properties and the resting potential were also measured. These findings of a RMP being more negative in the condition of ITS 1% + GDNF for 15 days as compared to the cells cultured in complete medium (growth medium, DMEM/F12 + 10% FBS) suggest that the cells are differentiating toward a more mature neural phenotype. The increase in capacitance (Cm) under differentiation conditions could be interpreted as the membrane charging/discharging more slowly, slowing signal propagation, and the increase in input resistance (Rin) corresponds to smaller cells that produce larger voltage changes for a given current. The reduced cell size observed under differentiated conditions further supports their neuronal-fate lineage induction stage compared to cells grown in complete medium with 10% FBS. When hDPSCs differentiate, they undergo morphological changes that make them smaller and thinner [26,75]. Although hDPSCs cultured in ITS 1% + GDNF for 15 days were more discreetly differentiated than those cultured in complete medium 10% FBS, they were not fully differentiated because they could not trigger action potentials. Furthermore, the resting membrane potential remained in the corresponding values of mesenchymal stem cells, such as the hDPSCs phenotype (−20 mV to −40 mV) [49,50].

Our results are consistent with those reported by Cortés et al. (2016) [76] regarding relation to GDNF effects on embryonic stem cells. They found that GDNF promotes neuronal differentiation by increasing the yield of differentiated motor neurons and improving their electrophysiological maturation, thus influencing both commitment and survival during the differentiation process of embryonic stem cells [76]. Furthermore, Bonafina et al. (2018) reported that GDNF promotes neuronal differentiation of cortical neural precursors by inhibiting their self-renewal capacity, particularly in the presence of low concentrations of FGF2, and enhances dendritic spine formation towards mitotic neurons, indicating its essential role in cortical development [77]. Regarding the mechanisms of action of GDNF, it has been reported that GDNF binds to the GFRα1 receptor, which is crucial for its signaling pathway. This interaction is enhanced by TGF-β, which facilitates GDNF signaling by localizing GFRα1 to the cell surface [75]. It has been proposed that the inhibition of self-renewal in cortical neural precursors is due to the GDNF/GFRα1 complex, which promotes differentiation even in the presence of growth factors such as FGF2 [77]. Likewise, knockout of GDNF in a Zebrafish model showed that its depletion leads to reduced dopaminergic neuron numbers and affects transcription factors related to neuron fate specification, indicating its crucial role in neuronal differentiation [78]. Further experiments are needed to clarify the mechanisms of action of the trophic factor GDNF on dental pulp stem cell differentiation, induction of neuronal fate, and possibly progress to neuronal maturation. In this respect, experiments such as pharmacological inhibition of GDNF signaling or siRNA-mediated targeting of GDNF receptors and signaling pathways could clarify role of this growth factor in hDPSCs’ neuronal-like differentiation.

Our study confirmed that hDPSCs naturally express immature neural markers in vitro. Under special permissive conditions, these cells must acquire more mature neuronal traits and exhibit markers beyond βIII Tubulin (Tuj-1), GFAP, and Nestin, such as NeuN [53,79]. Therefore, it is crucial to induce neural differentiation to its final stage of maturation to increase its therapeutic potential. Our results indicate that hDPSCs cultured in the presence of GDNF in a chemically defined medium differentiated into a neuronal phenotype, as evidenced by the reduction in the expression of the mesenchymal markers CD105 and CD73 and the increased expression of Nestin and β-III Tubulin (Tuj-1). After longer in vitro exposure to this growth factor (at 35 days in vitro), the more mature neuronal marker NeuN was expressed, correlating with more mature passive membrane properties. The efficacy of GDNF as a neurogenic factor depends on the concentration used, which can promote survival, differentiation, and anti-apoptotic activity. Concentrations between 12.5 and 100 ng/mL of GDNF neurotrophic factor have been reported to induce morphological changes, and increase cell survival, maintenance, and neuronal induction in MSCs [80]. GDNF is a significant neurotrophic factor that actively participates in the differentiation of MSCs into neuronal cells [81,82]. Investigations have shown that the combined action of GDNF and neurotrophin-3 (NT-3) stimulates the expression of neuronal markers such as MAP-2, Nestin, and Neuron Specific Enolase (NSE) [14,83]. By virtue of these characteristics and its ability to induce neuronal differentiation in MSCs, GDNF presents itself as a valuable alternative for the study and therapeutic implications of various neurological pathologies [84,85].

The hDPSCs differentiation protocol described herein is presented as a novel approach compared to other previously established methods (Table 4), as it uses a fully chemically defined medium free of fetal bovine serum (FBS): xeno-free. Unlike other protocols that rely on complex media with variable biological components, such as B27 or N2, or that only partially eliminate FBS (Table 4), this protocol provides greater reproducibility and consistency in the results by eliminating the variability associated with animal-derived serum. This not only simplifies medium preparation but also reduces operating costs and improves protocol standardization, facilitating its replication in different laboratories. Furthermore, precise control of growth factors ensures efficient and reproducible neuronal differentiation. One difference between this study and previous work in a similar area is the protocol described by Fatima et al. [86], which used a minimal concentration of FBS without specifying the exact composition of the medium. Our protocol features a fully defined formulation, eliminating any uncertainty regarding the compounds present in the media and ensuring that the differentiation effects are exclusively due to the growth factors added, constituting a more controlled, efficient, and clinically applicable approach to neuronal differentiation, and offering a solid basis for future therapeutic applications in regenerative medicine.

Further studies are warranted to explore the therapeutic potential of hDPSCs in the treatment of various neurodegenerative diseases.

## 5. Conclusions

This study aimed to establish a novel protocol for differentiating hDPSCs into a more mature neural phenotype. Our results confirm that hDPSCs can undergo neuronal commitment, as evidenced by key morphological and electrophysiological characteristics, as well as by the expression of mature neuronal markers. Despite this promising progress, obtaining a completely functional, differentiated culture remains an ongoing challenge.

Here, we propose an improved protocol in which a preliminary differentiation approach for hDPSCs, derived from a single donor, is associated with partial neuronal-like differentiation under chemically defined conditions. Prolonged GDNF exposure (15–35 DIV) in this chemically defined medium promoted hDPSCs differentiation into a partially mature neuronal phenotype, as evidenced by the expression of NeuN (a terminal neuronal differentiation marker) and altered electrophysiological membrane properties.

Our findings also align with the international criteria for mesenchymal stem cell (MSC) classification [12,23], confirming that dental pulp stem cells (hDPSCs) express canonical MSC markers (CD105, CD90, CD73, and CD13) while lacking hematopoietic markers (CD45, CD34, CD31, and CD14). Additionally, hDPSCs exhibited proliferative activity (Ki67) and basal expression of neural markers (Nestin, βIII-tubulin, and GFAP), consistent with their neural crest origin. Notably, prolonged exposure (15–35 days) to GDNF in a chemically defined medium induced hDPSCs to adopt a more mature neuronal phenotype, characterized by morphological changes, upregulation of NeuN, and alteration of passive membrane properties.

These findings highlight the potential of hDPSCs as promising tools for regenerative medicine and disease modeling, particularly in neurodegenerative disorders. However, further research is required to fully characterize hDPSC subpopulations using predictive marker profiles and validate their therapeutic utility through standardized differentiation protocols, functional assays, and electrophysiological studies. Such efforts will be critical for translating hDPSC-based therapies into clinical applications and disease modeling, as the fully chemically defined medium proposed in the present study could facilitate the standardization and reproducibility of hDPSC differentiation protocols.

## Figures and Tables

**Table 1:** PCR reaction components.

5× Taq Reaction Buffer BIOLINE	4 μL
cDNA from hDPSCs	20–50 ng
Forward Primer (0.1 μg/μL) (Table 2)	1 μL
Reverse Primer (0.1 μg/μL) (Table 2)	1 uL
DNA polymerase BIOLINE	0.3 μL
H_2_O sterile for molecular biology	For 20 μL
Total reaction	20 μL

**Table 2:** Primer sequences.

Mesenchymal Phenotype		
**Primer**	Sequence 5′-3′	Amplified Size
CD105_F	TGCACTTGGCCTACAATTCCA	105 pb
CD105_R	AGCTGCCCACTCAAGGATCT	
CD73_F	GCCTGGGAGCTTACGATTTTG	196 pb
CD73_R	TAGTGCCCTGGTACTGGTCG	
CD13_F	GACGCTGAGACCGTACCTC	201 pb
CD13_R	TCAGTCTTGTCAATGTCGGGG	
CD146_F	GGACCTTGAGTTTGAGTGG	479 pb
CD146_R	CAGTGGTTTGGCTGGAGT	
CD166_F	AACATGGCGGCTTCAACG	630 pb
CD166_R	GACGACACCAGCAACGAG	
CD31_F	GGTCTTGTCGCAGTATCAG	355 pb
CD31_R	ATGGCAATTATCCGCTCT	
**Neural Phenotype**		
**Primer**	Sequence 5′-3′	Amplified Size
NESTIN_F	CGCTCGGGAGAGTCGCTT	215 pb
NESTIN_R	CCAGTTGCTGCCCACCTTC	
TUB3_F	ACGCATCTCGGAGCAGTT	125 pb
TYB3_R	CGGACACCAGGTCATTCA	
GFAP_F	CCTCTCCCTGGCTCGAATG	161 pb
GFAP_R	GGAAGCGAACCTTCTCGATGTA	
NEUN_F	CCAAGCGGCTACACGTCTC	191 pb
NEUN_R	CGTCCCATTCAGCTTCTCCC	
GAPDH_F	CAACGGCACAGTCAAGG	717 pb
GAPDH_R	AAGGTGGAAGAGTGGGAG	

**Table 3:** Antibodies.

Mesenchymal Phenotype			
**Antibody**	Company	Catalog	Dilution (WB, IF)
CD105	Genetex (GeneTex, Inc., Irvine, CA, USA)	GTX100508	(1:1000) (1:500)
CD73	Genetex	GTX101140	(1:1000) (1:500)
CD13	Genetex	GTX102788	(1:1000) (1:500)
**Neural Phenotype**			
**Antibody**	Company	Catalog	Dilution
Nestin	Genetex	GTX30671	(1:1000) (1:500)
GFAP	Sigma Aldrich (MilliporeSigma, Burlington, MA, USA)	G3893	(1:1000) (1:500)
βIII-Tubulin	Genetex	GTX5078	(1:1000) (1:500)
NeuN	Genetex	GTX30773	(1:1000) (1:500)

**Table 4:** Comparative conditions used to differentiate hDPSCs into a neuronal phenotype.

Author	Differentiation Medium	Type of Culture	Differentiation Treatment Time	Markers Expressed
Pardo-Rodríguez et al., 2025 [79]	21–60 days to neural differentiation media composed by human Neurocult basal media supplemented with human Neurocult differentiation supplement (#05752, Stem Cell Technologies), 2% B-27 with vitamin A (#17504044, Thermo Fisher), 2 mM GlutaMAX (#11500626, Fischer Scientific), 100 U/mL penicillin and 150 mg/mL streptomycin (#11528876, Gibco, Invitrogen, Carlsbad, CA, USA). In some cases, 10 μM retinoic acid (RA) (#554720, AldrichMilliporeSigma, Burlington, MA, USA) and one hour pulses every two days of 40 mM potassium chloride (KCl) (#141494, PanReac AppliChem, Barcelona, Spain) Extra Pulse of Differentiation 10 uM of RA + 40 mM KCl	Spheres and Monolayer	21–60 days	Neural Progenitor Markers: Nestin, GFAP, S100β, p75NTR. Neuronal Maturity Markers: Doublecortin (immature), NeuN, MAP2, Ankyrin-G (mature). Synaptic Markers: vGLUT2 and Synapsin-I (presynaptic), glutamatergic, and GABAergic receptors (postsynaptic). Functional Ion Channels: TTX-sensitive Na+ and K+ channels enabling real action potentials.
Gervois et al., 2015 [53]	Neurobasal medium supplemented with 100 U/mL penicillin, 100 μg/mL streptomycin, 2 mM l-glutamine, 2% B27, 1% N2, 1 mM dbcAMP, and 30 ng/mL NT-3. The maturation medium was changed every 2 weeks, and the cells were maintained under maturation-promoting conditions for 4 weeks.	Neurospheres	28 days	NCAM, Synapsin I, GFAP, NeuN, MAP-2 and BDNF, NGF, and VEGF secretion were enhanced following neurogenic differentiation compared to that in non-differentiated hDPSCs.
Li et al., 2019 [87]	Protocol 1: MEM-alfa 10 ng/mL bFGF,10 μM forskolin,25 mM KCl, 2 mM valproic, acid 5 μg/mL insulin. Protocol 2: Induction DMEM-F12, 2% B27, 20 ng/mL EGF20 ng/mL bFGF. Maduration: NB 2 mM L-glutamine 2% B271% N21 mM dbcAMP30 ng/mL NT3	Spheres and Monolayer	7, 21 and 35 days	βIII-tubulin (Tuj-1), GFAP, MAP-2, NFM, pan-Nav (voltage-gated sodium channels (Nav)), GAD67, Nav1.6, NF1, NSE, PSD95 Only neurosphere-mediated method yielded electrophysiological properties of functional neurons.
Pisciotta et al., 2018 [88]	DMEM-F12, 2 mM L glutamin, 100 U/mL penicillin, 100 µg/mL streoptomycin, 2% B27 supplement, 20 ng/mL EGF, 20 ng/mL b-FGF, N2 100 ng/mL hNGF 10 ng/mL BDNF, 0.5 µM RA	Spheres and Monolayer	21 days	MAP-2, βIII-tubulin (Tuj-1), Nestin
Fatima et al., 2017 [86]	0.05 uM RA, 2% de SFB, human neural differentiation medium (Stem cell Technologies, Vancouver, BC, Canada)	Neurospheres and monolayer	21 days	04, GFAP, βIII-tubulin (Tuj-1), Nestin, NCAM
Ullah et a., 2016 [47]	Neurobasal A, 1× B27, 1% penicillin–streoptomycin, 20 ng/mL EGF y 30 ng/mL bFGF	Monolayer	21 days	βIII-tubulin (Tuj-1), NF-M, MAP-2, NGF, Tau, SYP, Synapsin

## Data Availability

The original contributions presented in this study are included in the article. Further inquiries can be directed to the corresponding author (mcardenasaguayo@unam.mx).

## Supplementary Materials

The following supporting information can be downloaded at: https://www.mdpi.com/article/10.3390/cells15100953/s1, Figure S1. Complete hDPSCs expression profile. The expression of cell markers was determined using flow cytometry. The results represent the number of positive cells for each marker.

## Abbreviations

The following abbreviations are used in this manuscript.

MSCs	Mesenchymal Stem Cells
hDPSCs	Dental Pulp Stem Cells
BDNF	Brain-derived Neurotrophic Factor
GDNF	Glial-derived Neurotrophic Factor
ITS	Insulin Transferrin Selenium Chemical defined medium
