# Supplementary material for: Chemically Defined Medium Enables GDNF-Driven Early Neuronal-like Phenotype of Human Dental Pulp Stem Cells
Source: Cells. 2026 May 21;15(10):953. doi: 10.3390/cells15100953 (PMC13204485; doi:10.3390/cells15100953)
Supplement: Supplementary file 1 [file cells-15-00953-s001.zip › cells-4248550-supplementary.pdf]

Marker	Percentage
CD90 PE Cy5	100
CD105 PE	25.3
CD73 PE Cy7	100
CD13 APC	99.7
HLA-ABC FITC	80
HLA-DR PE	0.36
CD45-PE	0.47
CD34-APC	1.05
CD31-FITC	0.83
CD14-FITC	0.56

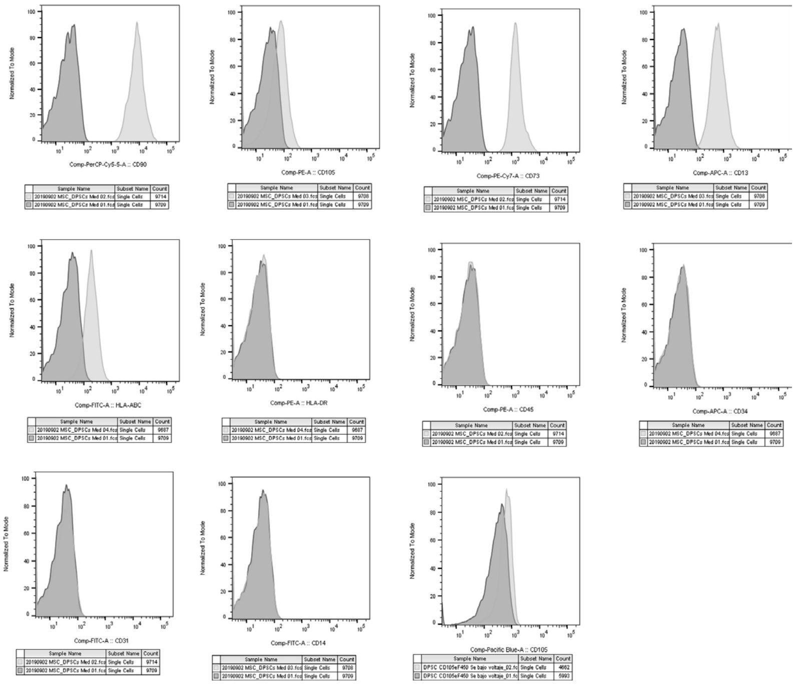

Figure S1 Complete hDPSCs expression profile. The expression of cell markers was determined using flow cytometry. The results represent the number of positive cells for each marker.
